# Association between subthreshold depression and self-care behaviors in people with type 2 diabetes: a systematic review of observational studies

**DOI:** 10.1186/s13643-020-01302-z

**Published:** 2020-02-29

**Authors:** Monika Shrestha, Ashley Ng, Amal Al-Ghareeb, Fatimah Alenazi, Richard Gray

**Affiliations:** 1 School of Nursing and Midwifery, La Trobe University, Melbourne, Australia; 2 Global Institute for Interdisciplinary Studies (GIIS), Kathmandu, Nepal; 3 Department of Dietetics, Human Nutrition and Sport, La Trobe University, Melbourne, Australia; 4 Melbourne, Australia; 5 University of South Australia, Adelaide, Australia; 6 University of Essex, Colchester, Essex UK

**Keywords:** Subthreshold depression, Depression, Self-care behavior, Self-management, Type 2 diabetes, Systematic review

## Abstract

**Background:**

Self-care behaviors in people living with type 2 diabetes are important to achieving optimal glycemic control. Major depression in type 2 diabetes is associated with decreased adherence to self-care behaviors. The association between subthreshold depression and self-care behaviors, however, has not previously been systematically reviewed. The objective of this review is to determine the association between subthreshold depression and self-care behaviors.

**Methods:**

A systematic search was performed in five electronic databases that included MEDLINE, EMBASE, PsycINFO, Emcare, and CINAHL. Any observational studies in adults with type 2 diabetes, investigating the association between subthreshold depression and any self-care behaviors, were included in the review. Qualitative studies, review articles, and gray literature were excluded. Two reviewers independently completed the title and abstract and full-text screening, appraised the study quality, and extracted the data. A third reviewer resolved any discrepancies between the reviewers if needed. Included articles were critically appraised using the Joanna Briggs Institute (JBI) Critical Appraisal Checklist. Meta-analyses were not conducted because criteria for conducting such analyses were not met.

**Results:**

A total of 6408 articles were identified through the database searching. After the abstract and full-text review, two articles met the inclusion criteria. One of the included study was cross-sectional while the other was a longitudinal study. Both studies showed inconsistent findings in the association between subthreshold depression and self-care behaviors. Important risks of bias were identified in the included studies.

**Discussion:**

The evidence from the two included studies on a possible association between subthreshold depression and self-care behaviors in adults with type 2 diabetes was not consistent and potentially biased. Our review established a gap in knowledge and suggests that further high-quality studies are needed to examine the association between subthreshold depression and self-care behaviors in people with type 2 diabetes.

**Systematic review registration:**

PROSPERO CRD42018116373

## Background

The global prevalence of diabetes is rapidly escalating as a result of several factors including urbanization, economic development, population aging, unhealthy eating habits, and sedentary lifestyles [1]. Over the past two decades, the number of people living with type 2 diabetes (T2D) worldwide has more than doubled [2]. International Diabetes Federation estimated that 1 in 11 adults aged 20–79 years (463 million) were living with T2D in 2019, and by 2045, this number is projected to rise to 700 million [3].

Type 2 diabetes, like other chronic diseases, is associated with an increased risk of comorbid health problems, including depression. Authors of a meta-analysis based on 248 studies and 83,020,812 participants reported that 28% of adults with T2D had a co-occurring depressive disorder [4]. People with T2D are twice as likely to develop depression, compared with the general population [5–7]. Comorbid depression in people with T2D is generally associated with poorer health outcomes including sub-optimal glycemic control [8–10], higher rates of diabetes-related complications [11, 12], poorer quality of life [13, 14], and increased risk of mortality [15, 16]. There is also evidence of an association between T2D, depression, and higher health care costs [17].

The association between subthreshold depression and T2D has not been extensively studied. Subthreshold depression occurs when an individual experiences depressive symptoms that do not meet the diagnostic threshold for a major depressive disorder (MDD) with respect to frequency, severity, and/or duration of symptoms [18, 19]. Common in adults, a systematic review of 19 studies estimated the prevalence of subthreshold depression between 3 and 10% in clinical and between 1 and 17% in community settings [19].

In some studies of people with T2D, the prevalence of subthreshold depression has been reported to be more common than MDD [20–22]. Like major depression, subthreshold depression is also associated with a higher rate of diabetes-related complications [11, 13], disability [11], and mortality [11, 23, 24]. There is some indicative evidence that subthreshold depression may be associated with an increase in HbA1c [25]. Further, subthreshold depression is associated with an increased risk of developing MDD. For example, a meta-analysis of 16 longitudinal cohort studies involving 67,318 participants identified that people with subthreshold depression were twice as likely than non-depressed people to develop MDD [26].

Effective management of diabetes requires adherence to self-care behaviors. In diabetes, self-care behaviors include monitoring diet, engaging in physical activity, routinely checking blood sugar levels, medication adherence, and active foot care [27]. Good adherence with self-care behaviors has shown to be associated with improved glycemic control [28–31], fewer diabetes-related complications [32], a decrease in the use of health services [33], and an improvement in quality of life [34].

People with T2D and MDD often demonstrate poor diabetes self-care behaviors. A meta-analysis by Gonzalez et al. that included 47 studies and 17,319 participants found a significant relationship between depression and non-adherence to self-care behaviors [35]. A systematic review of 27 studies and 7266 people with T2D observed that depression was associated with low adherence to diet and physical activity [36]. There may be an association between subthreshold depression and self-care behaviors in people with T2D. Our scoping searches of the literature did not identify any systematic reviews on this research topic. This systematic review aims to examine the association between subthreshold depression and self-care behaviors in adults with T2D.

## Methods

This systematic review is reported according to the Preferred Reporting Items for Systematic Reviews and Meta-Analyses (PRISMA) guidelines [37] (Additional file 1). The review was prospectively registered at the International Prospective Register of Systematic Reviews (PROSPERO) on 28th November 2018 (https://www.crd.york.ac.uk/PROSPERO/, registration number: CRD42018116373). A detailed summary of the review methods has been reported in our published protocol [38].

### Eligibility criteria

The review included studies that (i) were conducted in adults aged 18 years or over and diagnosed with T2D, (ii) were any observational studies (cross-sectional, case-control, or cohort), (iii) examined the association between subthreshold depression and any diabetes self-care behaviors, and (iv) were published in English. Studies were excluded if they were qualitative studies, review articles, and non-peer reviewed (gray) literature.

The self-care behaviors that were considered in the study were healthy eating, being physically active, monitoring of blood glucose, taking medication, not smoking, and foot care [27]. Currently, there is no agreed definition of subthreshold depression. Different terms—e.g., minor, subthreshold—are used to describe people with subthreshold depression [19]. For the purposes of our review, we defined subthreshold depression as minor, subthreshold, subclinical, subsyndromal depression, or a mood disorder that does not meet the diagnostic criteria for major depressive disorder [39].

### Data source and search strategy

The search strategy was developed in consultation with a medical librarian experienced in systematic review database searching. We conducted a systematic search of the literature in the following databases: MEDLINE, EMBASE, PsycINFO, Emcare, and CINAHL. Databases were searched using a combination of medical subject headings (MeSH) and keywords relating to “type 2 diabetes”, “depression”, and “self-care”. Boolean operators (“AND” and “OR”), proximity operators (“ADJ” and “N”), and truncation were incorporated into the search strategy as required to cater for the different use of terms. Search results were limited to English. Additional file 2 shows the Medline (ovid) search strategy used for this systematic review.

### Study selection

All the references identified were imported into Endnote X9 [40], a reference manager software program. Following the removal of duplicates in Endnote, citations were imported into Covidence systematic review software [41]. All studies were then assessed for eligibility in Covidence using a two-step process, (1) title and abstract screening and (2) screening of full texts. Two authors, MS and FA, applied the predetermined eligibility criteria to all the articles by screening titles and abstracts independently. All disagreements concerning inclusion or exclusion of papers were judged by a third author (AG). Full-text reading of the articles was again done independently by two authors (MS and FA). The final decision on the inclusion of the full-text articles was made after a discussion with the review team members.

### Data extraction

Two authors (MS and RG) independently extracted data from each of the included studies using the data extraction tool. A third author compared the authors’ data and resolved inconsistencies by referring to the full-text article and thorough discussion. The following data were extracted: citation, country of study, aim of the study, population characteristics (age, gender), study design (cross-sectional, case-control, or cohort) and setting (community or hospital), sample size, sample size calculation, sampling technique, data source (survey or secondary data), definition of subthreshold depression, measure used to examine subthreshold depression and self-care behaviors, analysis, confounder variables adjusted, and key observation of the study. There was no need to contact the authors of any studies for the study information.

### Quality appraisal

All the included studies were assessed for methodological rigor using the Joanna Briggs Institute (JBI) Critical Appraisal Checklist tool [42]. The JBI has separate checklists for cross-sectional (8 criteria), case-control (10 criteria), and cohort studies (11 criteria). Each component of the checklist was rated as yes, no, unclear, or not applicable. Two authors (MS and RG) independently evaluated the quality of each study, and disagreements were resolved by discussion within the review team.

### Data analysis

Due to a smaller number of studies and variability in the outcome measures, results could not be combined by meta-analysis. A narrative synthesis of the study was conducted. Tables and narrative summaries are used to present the study and participant characteristics and findings of the studies.

## Results

### Search results

**Fig. 1:**
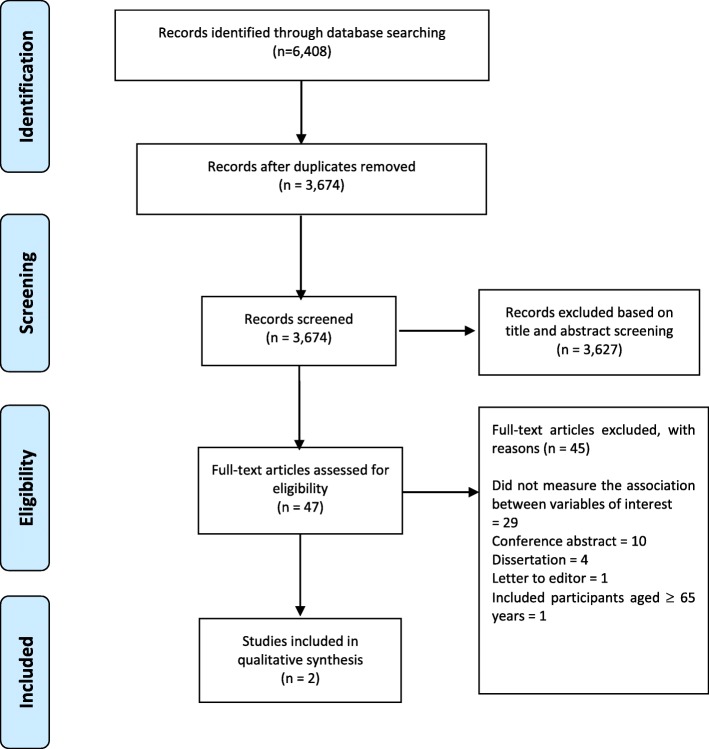
PRISMA flow chart showing the study selection process and search results

The details of the study selection process and search results are shown in Fig. .1. We identified a total of 6408 articles based on the systematic literature search in 5 databases: MEDLINE (*n* = 1147), EMBASE (*n* = 2955), PsycINFO (*n* = 447), Emcare (*n* = 1090), and CINAHL (*n* = 769). After removal of duplicates, the title and abstracts of 3674 articles were screened. Forty-seven studies were selected for a full-text review. Articles were excluded, generally, because study authors did not report on the association between subthreshold depression and self-care behaviors (refer to Additional file 3 for the excluded studies). Two studies that met the eligibility criteria were included in the review.

### Study characteristics

Both the included studies were hospital based. A longitudinal study of 866 primary care attenders in Germany was reported by Dirmaier et al. [43], and Shin et al. reported a cross-sectional survey of 103 outpatients in the USA [44]. Table 1 shows selected characteristics of the two included studies.

**Table 1:** Characteristics of included studies

Citation	Country of the study	Aim of the study	Study population characteristics	Study design	Study setting	Sample size/sample size calculation	Sampling technique	Data source	Definition of subthreshold depression	Measures used	Analysis	Confounder variables adjusted	Key observation
Dirmaier et al. [43]	Germany	To investigate the effect of depression on adherence to medical recommendation (concerning medication adherence and health behavior) and to investigate the effect of depression and adherence to medical recommendation (medication adherence and health behavior) on HbA1c	Type 2 diabetes patients aged 18 years or older; mean age, 65.7 years; female, 48.8%	Longitudinal study	Hospital based in primary care	866/not specified	Random sampling	DETECT study of 55,518 patients	DSQ score between 5 and 7 indicated subthreshold depression	-Depression Screening Questionnaire (DSQ) -Medication non-adherence: one question -Non-adherence with health behavior: five item questionnaires	Logistic and linear regression	Gender, age, marital status, employment status, education, physical activity, BMI, smoking, drinking, duration of diabetes, and type of diabetes treatment	Subthreshold depression was associated with problems with health behavior whereas it was not associated with medication adherence.
Shin et al. [44]	USA	To examine whether problem-solving and diabetes self-care behaviors differed by depressive disorder diagnosis	Type 2 diabetes patients aged 18 years or older; mean age, 58 years; female, 59%	Cross-sectional study	Hospital based	103/not specified	All patients with type 2 diabetes seen in the clinic between 1st Feb 2011 and 30th June 2013.	Primary (data from patients attending Diabetes Center Clinics at John Hopkins)	DSM-IV criteria for minor depression	-PHQ-2 followed by SCID-I/NP -Summary of Diabetes Self-Care Activities (SDSCA)	ANOVA and multiple linear regression	Age, sex, and diabetes duration	Minor depression was not associated with neither the total score nor the subscale score (diet, exercise, blood sugar, foot care, medication) of self-care activities.

*SCID-I/NP* Structured Clinical Interview for the DSM-IV Axis I Disorders, non-patient edition

#### How was subthreshold depression determined?

The exposure, subthreshold depression, was determined using different procedures in the included studies. Dirmaier et al. classified participants as having subthreshold depression or depression if they had a Depression Screening Questionnaire (DSQ) score of between 5 and 7 or 8 or higher, respectively [43]. Shin et al. used a two-step process to make a depression diagnosis [44]. Initially, a screening questionnaire, the two-item version of the Patient Health Questionnaire (PHQ-2) [45], was completed and scored. Participants with a score of 3 or higher were assessed by a clinician using the Structured Clinical Interview for the Diagnostic and Statistical Manual of Mental Disorders-IV (DSM-IV) Axis I Disorders, non-patient edition [46]. Also, participants taking any antidepressant medication (for any reason) and those who already had a MDD diagnosis were interviewed by a researcher. Participants were determined to have minor or major depression if they met DSM-IV diagnostic criteria [47].

#### How were self-care behaviors determined?

In the Dirmaier et al. study, six items in the questionnaire were used to determine self-care behaviors [43]: A single item was used to determine medication non-adherence and five items to determine non-adherence to other health behaviors (diet (2 items), smoking, alcohol, and physical activity). Shin et al. [44] used the Summary of Diabetes Self-Care Activities (SDSCA) [48] to determine participants’ level of self-care behaviors during the past 7 days. Self-care behaviors were assessed across five domains, which included diet, exercise, foot care, blood glucose testing, and medication taking. The overall self-care behavior was calculated by adding up the scores of all the domains.

### Participant characteristics

Participant characteristics are shown in Table 1. Included studies involved 968 adults (aged 18 years and above) participants. In both studies, males and females were equally represented [43, 44]. Participants in the Dirmaier et al. study [43] were older than those in Shin et al. study [44]. Mean diabetes duration was around 10 years in both studies [43, 44].

### Quality appraisal

Quality appraisal of included studies is shown in Table 2. Since none of the included studies were of a case-control study design, the JBI Critical Appraisal Checklist tool for cross-sectional and cohort studies was used. None of the studies was excluded based on their quality appraisal.

**Table 2:** Quality appraisal of included studies

Study		Cohort study	Yes	No	Unclear	Not applicable
Dirmaier et al. [43]	1.	Were the two groups similar and recruited from the same population?	X			
	2.	Were the exposures measured similarly to assign people to both exposed and unexposed groups?	X			
	3.	Was the exposure measured in a valid and reliable way?	X			
	4.	Were confounding factors identified?	X			
	5.	Were strategies to deal with confounding factors stated?	X			
	6.	Were the groups/participants free of the outcome at the start of the study (or at the moment of exposure)?				X
	7.	Were the outcomes measured in a valid and reliable way?		X		
	8.	Was the follow-up time reported and sufficient to be long enough for outcomes to occur?	X			
	9.	Was follow-up complete, and if not, were the reasons to loss to follow-up described and explored?			X	
	10.	Were strategies to address incomplete follow-up utilized?			X	
	11.	Was appropriate statistical analysis used?	X			
Shin et al. [44]	1.	Were the criteria for inclusion in the sample clearly defined?	X			
	2.	Were the study subjects and the setting described in detail?			X	
	3.	Was the exposure measured in a valid and reliable way?	X			
	4.	Were objective, standard criteria used for measurement of the condition?	X			
	5.	Were confounding factors identified?			X	
	6.	Were strategies to deal with confounding factors stated?	X			
	7.	Were the outcomes measured in a valid and reliable way?	X			
	8.	Was appropriate statistical analysis used?	X			

In the study by Dirmaier et al., 3 of 11 items were rated as potential sources of bias [43]. Of particular concern, key variables were determined using measures that were developed specifically for the study—e.g., medication non-adherence—despite validated measures being available. The flow of participants through the study is not clear and that authors do not state strategies to address the incomplete follow-up.

Several potentially important sources of bias were identified in the Shin et al. study [44]. Six items were rated low and two unclear risk of bias. The most important source of potential bias was related to confounding, as authors adjusted for three confounders (age, sex, and diabetes duration) in their analysis. Other potential confounders that may have impacted the observed association have not been addressed. The information about the setting of the study is not sufficiently detailed. Although the authors articulated clear hypotheses, the authors did not report a sample size calculation; consequently, it cannot be determined if the sample size was appropriate to test the expressed hypotheses.

Both the studies have used self-report tools to measure self-care behaviors [43, 44]. The study by Dirmaier et al. used a single item to measure medication adherence [43]. Health behavior was measured using a tool that was not validated, and the items specific to diabetes self-care behavior such as blood glucose testing and foot care were not included to measure total health behavior [43].

Based on these assessments, the overall methodological quality of included studies was judged to be potentially biased because of a number of reasons that included limitation in the research design, lack of use of validated measure, inappropriate confounder adjustments, and a small sample size.

### Association between subthreshold depression and self-care behaviors

Dirmaier et al. reported that subthreshold depression was associated with non-adherence to health behavior over a period of 12 months follow-up [43]. The association was retained (*β* = 1.01, CI 0.62–1.40, *p* < 0.001) after adjusting for gender, age, marital and employment status, education, physical activity, BMI, smoking, drinking, duration of diabetes, and type of diabetes treatment. No significant association between subthreshold depression and medication non-adherence was observed.

Shin et al. reported that individuals with T2D and subthreshold depression scored lower in their self-care behavior as compared with the group without depression [44]. However, there was no significant association between subthreshold depression and overall self-care behavior or individual self-care domains (diet, exercise, blood sugar, foot care, and medication).

## Discussion

This is the first review to systematically appraise and synthesize studies examining the association between subthreshold depression and diabetes self-care behaviors in adults with T2D. Our review included 2 studies involving 968 participants. Important potential sources of bias were identified in both included studies. One study showed a significant and the other a non-significant association between subthreshold depression and self-care behaviors. Based on these findings and the various quality issues identified in both studies, we concluded that the evidence on the association between subthreshold depression and self-care behaviors in people with T2D was inconsistent and potentially biased.

In people with T2D and MDD, there is clear evidence of an association with adherence to self-care behaviors. It is perhaps surprising that so few studies have tested this association in patients with T2D and subthreshold depression. Subthreshold depression is prevalent in T2D, affecting around 12% of patients [22]. Examining the association between subthreshold depression and self-care is perhaps an important area for further inquiry. Policymakers and researchers should prioritize further observational and experimental research in this area. If subthreshold depression is associated with decreased adherence to self-care behaviors, then this finding could become an important item for guidelines on the treatment and management of subthreshold depression in people with T2D. Effective management of subthreshold depression in people with T2D could prevent them from progressing into major depression.

This review has several limitations that need consideration. The review considered six domains of diabetes self-care behaviors that are often recommended for people with T2D: diet, physical activity, blood glucose monitoring, medication, foot care, and smoking. Other self-care behaviors, such as attending a diabetes education program and those recommended by the American Association of Diabetes Educators such as problem-solving, were not considered in this review [27]. A further limitation was the exclusion of the non-peer reviewed literature. We may have missed some of the important data by not having included gray literature in our review. However, as the validity of gray literature is difficult to be determined, this exclusion may be of less importance. We limited our search to the English language. It may be possible that studies published in other languages might have provided additional data, especially in a case such as ours, where the evidence is limited.

The included studies also have their own limitations that have been acknowledged by the authors. Both the studies are hospital based. Hence, the findings cannot be generalized to the community population with T2D. Similarly, the use of self-report tool in the studies may have overestimated the true adherence level as a result of social desirability bias and might have affected the result of the study. The findings from the study by Dirmaier et al. should be interpreted with caution as the study does not use a validated tool to measure self-care behaviors.

## Conclusions

This review identified two studies that examined the association between subthreshold depression and self-care behaviors in people with T2D. The evidence on this association was found to be inconsistent and potentially biased. High-quality research studies are necessary to further explore this association which may subsequently help in planning interventions to improve self-management of people with T2D.

## Supplementary information

**Additional file 1.**

**Additional file 2.**

**Additional file 3.**

## Data Availability

All the data supporting the conclusion of this review is included within the article and its additional files.

## Abbreviations

DSM-IV: Diagnostic and Statistical Manual of Mental Disorders-IV
DSQ: Depression Screening Questionnaire
JBI: The Joanna Briggs Institute
MDD: Major depressive disorder
PHQ: Patient Health Questionnaire
PRISMA: Preferred Reporting Items for the Systematic Reviews and Meta-Analyses
PROSPERO: International Prospective Register of Systematic Reviews
SDSCA: Summary of Diabetes Self-Care Activities
T2D: Type 2 diabetes
