# Supplementary material for: Association between subthreshold depression and self-care behaviors in people with type 2 diabetes: a systematic review of observational studies
Source: Syst Rev. 2020 Feb 29;9:45. doi: 10.1186/s13643-020-01302-z (PMC7049390; doi:10.1186/s13643-020-01302-z)
Supplement: Supplementary file 3 — Additional file 3. [file 13643_2020_1302_MOESM3_ESM.docx]

**List of excluded studies after full text review organized under sub-heading that states reason for exclusion (n=45)**

**Did not measure the association between the variables of interest**

1. Adam JE. Depressive symptoms, self-efficacy and adherence in patients with type 2 DM. Communicating Nursing Research. 2011; 44:296.
2. Aikens JE. Prospective associations between emotional distress and poor outcomes in type 2 diabetes. Diabetes Care. 2012;35(12):2472-8.
3. Al-Amer R, Ramjan L, Glew P, Randall S, Salamonson Y. Self-Efficacy, Depression, and Self-Care Activities in Adult Jordanians with Type 2 Diabetes: The Role of Illness Perception. Issues in Mental Health Nursing. 2016;37(10):744-55.
4. Al-Hayek AA, Robert AA, Alzaid AA, Nusair HM, Zbaidi NS, Al-Eithan MH, et al. Association between diabetes self-care, medication adherence, anxiety, depression, and glycemic control in type 2 diabetes. Saudi Medical Journal. 2012;33(6):681-3.
5. Arteaga-Zarate G, Demarini-Olivares G, Torres-Slimming PA. Symptoms of anxiety and depression in type 2 diabetes: Associations with clinical diabetes measures and self-management outcomes in the norwegian HUNT study. Psychoneuroendocrinology. 2018;89:256.
6. Badawi G, Page V, Smith KJ, Gariepy G, Malla A, Wang J, et al. Self-rated health: A predictor for the three year incidence of major depression in individuals with Type II diabetes. Journal of Affective Disorders. 2013;145(1):100-5.
7. Browne JL, Nefs G, Pouwer F, Speight J. Depression, anxiety and self-care behaviours of young adults with Type 2 diabetes: results from the International Diabetes Management and Impact for Long-term Empowerment and Success (MILES) Study. Diabetic Medicine. 2015;32(1):133-40.
8. Ciechanowski PS, Katon WJ, Russo JE. Depression and diabetes: impact of depressive symptoms on adherence, function, and costs. Archives of Internal Medicine. 2000;160(21):3278-85.
9. Ciechanowski PS, Katon WJ, Russo JE, Hirsch IB. The relationship of depressive symptoms to symptom reporting, self-care and glucose control in diabetes. General Hospital Psychiatry. 2003;25(4):246-52.
10. Coelho CR, Zantut-Wittmann DE, Parisi MC. A cross-sectional study of depression and self-care in patients with type 2 diabetes with and without foot ulcers. Ostomy Wound Management. 2014;60(2):46-51.
11. Collins-McNeil JC, Holston EC, Edwards CL, Benbow D, Ford Y. Physical activity, depressive symptoms, and social support among African-American women with type 2 diabetes. Canadian Journal of Nursing Research. 2009;41(3):24-43.
12. Daniele TM, Bruin VM, Oliveira DS, Pompeu CM, Forti AC. Associations among physical activity, comorbidities, depressive symptoms and health-related quality of life in type 2 diabetes. Arquivos Brasileiros de Endocrinologia e Metabologia. 2013;57(1):44-50.
13. Anonymous. Depressive symptoms are associated with self-care non-adherence in type 2 diabetics. Nature Clinical Practice Endocrinology and Metabolism. 2007;3(10):677.
14. Daniele TM, de Bruin VM, e Forte AC, de Oliveira DS, Pompeu CM, de Bruin PF. The relationship between physical activity, restless legs syndrome, and health-related quality of life in type 2 diabetes. Endocrine. 2013;44(1):125-31.
15. Gaitonde P, Shaya FT. Relationship between depression, self-care behaviors, and treatment success among older Medicare beneficiaries with type 2 diabetes. Journal of Pharmaceutical Health Services Research. 2016;7(4):241-5.
16. Gonzalez JS, Safren SA, Cagliero E, Wexler DJ, Delahanty L, Wittenberg E, et al. Depression, self-care, and medication adherence in type 2 diabetes: relationships across the full range of symptom severity. Diabetes Care. 2007;30(9):2222-7.
17. Gonzalez JS, Safren SA, Delahanty LM, Cagliero E, Wexler DJ, Meigs JB, et al. Symptoms of depression prospectively predict poorer self-care in patients with Type 2 diabetes. Diabetic Medicine. 2008;25(9):1102-7.
18. Hernandez R, Ruggiero L, Prohaska TR, Chavez N, Boughton SW, Peacock N, et al. A Cross-sectional Study of Depressive Symptoms and Diabetes Self-care in African Americans and Hispanics/Latinos With Diabetes: The Role of Self-efficacy. Diabetes Educator. 2016;42(4):452-61.
19. Johnson ST, Al Sayah F, Mathe N, Johnson JA. The relationship of diabetes-related distress and depressive symptoms with physical activity and dietary behaviors in adults with type 2 diabetes: A cross-sectional study. Journal of Diabetes & its Complications. 2016;30(5):967-70.
20. Kalsekar ID, Madhavan SS, Amonkar MM, Makela EH, Scott VG, Douglas SM, et al. Depression in patients with type 2 diabetes: impact on adherence to oral hypoglycemic agents. Annals of Pharmacotherapy. 2006;40(4):605-11.
21. Kendzor DE, Chen M, Reininger BM, Businelle MS, Stewart DW, Fisher-Hoch SP, et al. The association of depression and anxiety with glycemic control among Mexican Americans with diabetes living near the U.S.-Mexico border. BMC Public Health. 2014;14:176.
22. Koopmans B, Pouwer F, de Bie RA, van Rooij ES, Leusink GL, Pop VJ. Depressive symptoms are associated with physical inactivity in patients with type 2 diabetes. The DIAZOB Primary Care Diabetes study. Family Practice. 2009;26(3):171-3.
23. Lin EH, Katon W, Von Korff M, Rutter C, Simon GE, Oliver M, et al. Relationship of depression and diabetes self-care, medication adherence, and preventive care. Diabetes Care. 2004;27(9):2154-60.
24. Lunghi C, Zongo A, Moisan J, Gregoire JP, Guenette L. The impact of incident depression on medication adherence in patients with type 2 diabetes. Diabetes & Metabolism. 2017;43(6):521-8.
25. Messier L, Schmitz N, Elisha B, Garièpy G, Malla A, Lesage A, et al. Lifestyle and care indicators in individuals with major, minor and no depression: a community-based diabetes study in Quebec. Canadian Journal of Diabetes. 2011;35(1):22-30.
26. Nguyen AL, Green J, Enguidanos S. The relationship between depressive symptoms, diabetes symptoms, and self-management among an urban, low-income Latino population. Journal of Diabetes & its Complications. 2015;29(8):1003-8.
27. Pagoto SL, Ma Y, Bodenlos JS, Olendzki B, Rosal MC, Tellez T, et al. Association of depressive symptoms and lifestyle behaviors among Latinos at risk of type 2 diabetes. Journal of the American Dietetic Association. 2009;109(7):1246-50.
28. Wu SF, Huang YC, Liang SY, Wang TJ, Lee MC, Tung HH. Relationships among depression, anxiety, self-care behaviour and diabetes education difficulties in patients with type-2 diabetes: a cross-sectional questionnaire survey. International Journal of Nursing Studies. 2011;48(11):1376-83.
29. Zhang J, Xu CP, Wu HX, Xue XJ, Xu ZJ, Li Y, et al. Comparative study of the influence of diabetes distress and depression on treatment adherence in Chinese patients with type 2 diabetes: A cross-sectional survey in the People's Republic of China. Neuropsychiatric Disease and Treatment. 2013;9:1289-94.

**Conference abstracts**

1. Grinberg K, Amzaleg M, Panadha M, Mahamid R. The relationship between type 2 diabetes patients and depression. Diabetes Technology and Therapeutics. 2018;20 (Supplement 1):A75.
2. Hernandez R, Ruggiero L, Prohaska T, Chavez N, Peacock N, Nouwen A. The association between depressive symptoms and diabetes self-care in low income African American and Latino patients with type 2 diabetes in a primary care setting. Diabetes. 2013;1):A197-A8.
3. Johnson ST, Al Sayah F, Mathe N, Johnson JA. Depressive symptoms and diabetes distress associated with physical inactivity and poorer dietary practices in adults with type 2 diabetes. Diabetes. 2015;1):A222.
4. Lynch EB, Karavalos K, Avery E, Fogelfeld L. Depressive symptoms decrease medication adherence and general diet adherence in African-American adults with type 2 diabetes. Diabetes. 2015;1):A220.
5. Murray LV, Wilmot EG, Davies MJ, Yates T, Khunti K. Depression, glycaemic control, and physical activity in a multi-ethnic population screened for type 2 diabetes. Diabetologia. 2010;1):S392-S3.
6. Yang P, Swardfager W, Oh PI, Thomas SG. Depressive symptoms predict effectiveness of exercise-based rehabilitation in people with type 2 diabetes. Canadian Journal of Diabetes. 2014;5):S13.
7. Pibernik-Okanovic M, Ajdukovic D, Metelko Z. Interaction between depressive symptoms and illness representations in predicting self-care behaviours in type 2 diabetic patients. Diabetologia. 2010;1):S393.
8. Rahe C, Khil L, Wellmann J, Baune B, Arolt V, Berger K. The relationship between symptom severity of major depressive disorder and lifestyle. European Journal of Epidemiology. 2016;31 (Supplement 1):S159.
9. Sperl-Hillen J, Beaton S, Parker E, Fernandes O, Von Worley A, Hanson A, et al. The relationship between depression and attitudes, self-efficacy, self-care adherence and A1C in patients with suboptimally controlled diabetes. Diabetes Conference: 70th Scientific Sessions of the American Diabetes Association Orlando, FL United States Conference Publication:. 2010.
10. Lunghi C, Zongo A, Moisan J, Grégoire J-P, Guénette L. 189 - The Association Between Depression and Medication Non-Adherence in Type 2 Diabetes: A Population-Based Cohort Study. Canadian Journal of Diabetes. 2016;40:S68-S.

**Dissertation**

1. Al-Amer R. Depression and self-care in Jordanian adults with diabetes: The POISE study. Dissertation Abstracts International Section C: Worldwide. 2018;75(1-C)
2. Elliott JL. The relationship of self-efficacy, depression, and monitoring to self-reported adherence in type 2 diabetes. Dissertation Abstracts International: Section B: The Sciences and Engineering. 2003;63(9-B):4416.
3. Hernandez R. Longitudinal relationship between depression and diabetes self-care in minorities with type 2 diabetes. Dissertation Abstracts International: Section B: The Sciences and Engineering. 2013;74(5-B(E))
4. Meehan DB. Cognitive functioning, comorbid depression, and medication adherence in adults with type 2 diabetes. Dissertation Abstracts International: Section B: The Sciences and Engineering. 2018;79(11-B(E))

**Letter to editor**

1. Egede LE, Echols CL, Richardson LK, Mueller M, Gebregziabher M. Depression, physical activity and glycemic control in adults with type 2 diabetes. General Hospital Psychiatry. 2009;31(3):299-300.

**Participants aged ≥65 years**

1. Gentil L, Vasiliadis HM, Berbiche D, Preville M. Impact of depression and anxiety disorders on adherence to oral hypoglycemics in older adults with diabetes mellitus in Canada. European Journal of Ageing. 2017;14(2):111-21.
